# Role of Garlic Usage in Cardiovascular Disease Prevention: An Evidence-Based Approach

**DOI:** 10.1155/2013/125649

**Published:** 2013-04-17

**Authors:** Waris Qidwai, Tabinda Ashfaq

**Affiliations:** Department of Family Medicine, Aga Khan University Stadium Road, P.O. Box 3500, Karachi 74800, Pakistan

## Abstract

*Introduction*. Rapidly growing prevalence of cardiovascular disease is a major threat for the developed as well as developing world warranting urgent need of intervention. Complementary and alternative medicines are gaining popularity among general population because of their safety profile and easy administration. Garlic, in particular, is considered to be one of the best disease-preventive foods because of its potent and widespread effects. This study was done to find out the role of garlic usage in cardiovascular disease prevention. *Methodology*. Major databases including Google, PubMed, MEDLINE, and Cochrane library view were used for the literature search. Clinical trials conducted on humans assessing role of garlic usage in cardiovascular disease prevention and the possible mechanisms responsible for such therapeutic actions were assessed. *Results*. Various clinical trials and meta-analyses conducted have shown positive impact of garlic in cardiovascular-disease prevention especially its effects on lipid levels; however, some contradictory results are also reported. Similarly, its effects on hypertension control, and platelet are also mild with limited data availability. The possible reason for these inconsistent results is the difference in preparations with diverse composition, variations in sulphur content present in different garlic preparations used, and methodological variations in subject recruitment, duration of study, dietary control and so forth. *Conclusion*. Garlic can be used as an adjuvant with lipid-lowering drugs for control of lipids, however, its role as a main therapeutic agent cannot be recommended and it is suggested that more meta-analyses using standardized preparations with a close watch on methodological shortfalls should be conducted to prove its role.

## 1. Introduction

The epidemic of cardiovascular disease is growing at an alarming pace throughout the world [1]. It is recognized as one of the leading causes of mortality worldwide causing more than 80% of deaths in low- and middle-income countries [2]. Cardiovascular disease refers to spectrum of illnesses that includes heart disease, vascular diseases of the brain, kidney, and peripheral arterial disease [3]. According to an estimate by World Health Organization, approximately 17.3 million people died from CVDs in 2008, representing 30% of all global deaths. Out of these deaths, 7.3 million occurred secondary to coronary heart disease and 6.2 million as a consequence of stroke [2]. It is anticipated that by 2020 cardiovascular diseases are predicted to be the major cause of morbidity and mortality in most developing nations around the globe [4]. Atherosclerosis and hypertension are measured as the major risk factor along with smoking, obesity, and sedentary life styles leading to increasing trend of this major threat [5].

Today, in this era of rapid advancement in medical technology, herbal or botanical preparations, commonly referred to as complementary and alternate medicine (CAM), approaches have gained lots of popularity in terms of health care maintenance, and a large number of population in the developing as well as developed world prefer to use (CAM) as a source of curative and preventive remedy for various illnesses [6, 7]. CAM is defined as a group of diverse medical and health systems practices and products that are not generally considered as part of conventional medicine [8]. According to 2007 National Health Interview Survey (NHIS) report, approximately 38% of US adults and 12% of children are using CAM in the past 12 months; lifetime prevalence of CAM use in the United States and worldwide has increased steadily since 1950 (9.10). Most common types of CAM therapies used were natural products, such as fish oil/omega 3, glucosamine, *Echinacea*, and flaxseed (17.7%), deep breathing (12.7%), meditation (9.4%), chiropractic and osteopathic (8.6%), and massage (8.3%), followed by yoga, diet-based therapies, progressive relaxation, guided imagery, and homeopathic treatment [9]. 

Among all these remedies, plants-based functional foods have gained lots of recognition throughout the world and it is believed that these natural substances have the potential to reduce the detrimental effect of a number of cardiovascular diseases and associated risk factors [10]. The probable reason for this rising trend is skeptic approach of general public towards conventional medicine due to fear of more side effects and increasing cost. This fact was further driven by the belief of increased safety profile and easy availability of plant-based natural products in comparison to orthodox medicine. Garlic has been used as a therapeutic agent for many illnesses over centuries as evident from various studies; however, its role in cardiovascular disease prevention is still questionable. This review was done to find out the efficacy of garlic in cardiovascular disease prevention through evidence-based approach by analyzing clinical trials and systematic review in the above mentioned area.

## 2. Garlic as a Potential Herb

Garlic (*Allium sativum*) has played an important dietary as well as medicinal role in human history. The role of garlic (*Allium sativum*) as a potential herb has been acknowledged for over 5000 years. Garlic and its various preparations are being readily consumed as a food and spice by various cultures for centuries [11]. It was also documented as a choice of medical therapy to combat many diseases among Egyptians [12]. Similarly, it is also considered as an imperative part of Indian traditional medicine, that is, Ayurveda, Tibbi and Unani, and so forth. In addition, it is also claimed to possess beneficial effects for the prevention of various aspects of cardiovascular disease including hypertension and dyslipidemia [13]. 

## 3. Garlic Composition

Garlic is available in many forms among these raw garlic and aqueous extract preparation is used more frequently. Allicin is the principal bioactive compound present in garlic and primarily contains sulphur as a main constituent which on break down gives garlic its characteristic odor. It is produced as a result of activation of alliinase enzyme after crushing or chopping of raw garlic. The enzyme Allinase is inactivated by heat leaving behind alliin as the main constituent present in the water extract of heat-treated garlic. The composition of garlic powder which is produced after dehydration and crushing is the same as raw garlic and alliinase activity is preserved; however, caution needs to be taken regarding temperature regulation as Allinase is inactivated if temperature exceeds beyond 60°C. Apart from Alliin, other important sulfur-containing compounds present in garlic homogenate include allyl methyl thiosulfonate, 1-propenyl allyl thiosulfonate, and *γ*-L-glutamyl-S-alkyl-L-cysteine. On an average, a garlic bulb contains up to 0.9% g-glutamylcysteines and up to 1.8% alliin [14].

## 4. AGE Preparation

Another important and extensively studied garlic preparation is aged garlic extract (AGE). This form is produced by storage of sliced raw garlic in 15–20% ethanol for 20 months. This process of storage leads to alteration in composition of the garlic extract, the odorous, harsh, and irritating compounds in garlic are converted naturally into stable and safe sulfur compounds with substantial loss of allicin activity and increased activity of new compounds, like S-allylcysteine (SAC), S-allylmercaptocysteine, and allixin [15]. SAC can be used for standardization because of its bioavailability property.

## 5. Garlic Oil

Garlic oil another important preparation is produced as a result of distillation process of raw garlic. Garlic essential oil is obtained by steam distillation of garlic. The essential oil content of garlic cloves is 0.2–0.5% and consists of a variety of sulfides, such as diallyl disulphide (DADS) and diallyl trisulfide (DATS) [16, 17]. All the water soluble contents including allicin are completely eliminated from the oil. Oil macerates were originally developed for use as condiments. Oil macerate products are made of encapsulated mixtures of whole garlic cloves ground into vegetable oil. This preparation contains allicin-decomposed compounds such as dithiins, ajoene and sulfides, residual amounts of alliin, and other constituents in garlic [16].

## 6. Garlic Powder

Garlic powder is primarily used as a flavoring agent for condiments and processed foods. Garlic cloves are sliced or crushed, dried, and ground into powder. The composition of garlic powder is the same as that of raw garlic; however, the proportions and amounts of various constituents differ significantly; that is, average content of alliin present in garlic is 0.8% however, raw garlic contains around 3.7 mg/gm of alliin [18]. 

## 7. Garlic and Cardiovascular Disease Prevention

Cardiovascular disease is one of the leading cause of morbidity and mortality worldwide. Role of garlic in cardiovascular disease prevention has been a topic of concerns for many years. Various observational and experimental studies done on animals showed encouraging results. However, these claimed benefits were not supported by evidence-based clinical studies. This fact prompted many researchers to conduct clinical trials in order to explore and address the efficacy and association of garlic with various aspects of cardiovascular disease. 

In recent years, garlic has been a focus of attention because of its potential role in the prevention of various aspects of cardiovascular disease [19, 20]. Evidence from numerous studies suggests that garlic works through various mechanisms to achieve this favorable effect including reduction of serum lipids and blood pressure levels, inhibition of platelet aggregation, and increasing fibrinolytic antioxidant activity. Majority of the studies reported have shown positive impact; however, few numbers of contradictory studies have [21, 22] made the role of garlic questionable especially with regards to its effects on lipid levels and hypertension. This review will critically examine the current scientific literature concerning claims of cardiovascular benefits from regular consumption of garlic or its preparations and the possible mechanisms responsible for such therapeutic actions. 

## 8. Methodology

This paper is based on a literature search of clinical trials and systematic reviews published from 1990–2012 to see the effect of Garlic on cardiovascular disease prevention. For this purpose multiple search engines including MEDLINE, PubMed, Google, and Cochrane library were used. Search was validated by other author.

## 9. Inclusion Criteria

All human studies (clinical trials) in English assessing the effect of garlic on cardiovascular disease prevention among patients with dyslipidemia, hypertension, or cardiovascular disease were included.

## 10. Exclusion Criteria

Studies conducted among animals were excluded. Theses, dissertations, unpublished data, and letter to editor were also excluded. 

A number of keywords were used for data searching including garlic and cardiovascular disease clinical trial, garlic hypertension and dyslipidemia, platelet aggregation, and clinical trial.

## 11. Garlic Role in Dyslipidemia

Dyslipidemia is documented as a major risk factor responsible for the development of atherosclerosis and cardiovascular disease [23]. Lipid abnormalities include high LDL-C (low-density lipoprotein cholesterol), high triglycerides and low HDL-C (high-density lipoproteins cholesterol) levels. Cholesterol present in *β*-lipoprotein (LDL) and pre-B-lipoprotein gets deposited into the blood vessels, while *α*-lipoprotein (HDL) helps to reduce serum cholesterol [24]. Impact of garlic on elevated lipid level is the most widely studied outcome of interest as evident from Table 1. Considerable evidence from the literature supports the invaluable role of garlic in the treatment of hypercholesterolemia through inhibition of cholestrol biosynthesis in the liver and also by inhibition of oxidation of low-density lipoproteins [25]. Dietary approach is the initial step in the management of dyslipidemia, and many people with dyslipidemia are using garlic as an alternative medicine to normalize their raised lipid levels. 

A number of randomized, controlled trials were carried out to see the effect of different preparation of garlic on lipid levels. In the early 1980s, a trial done [19] on human subject after ingestion of 40 gm garlic demonstrated significant reduction in total cholestrol and triglyceride levels. Similarly, one study conducted by Mader [20] in 1990 among patients suffering from dyslipidemia over a period of 16 weeks using 800 mg of garlic (standardized to 1.3% of Alliin) showed 12% reduction in serum cholestrol levels and 17% reduction in triglyceride levels in comparison to placebo; however, it was also noticed that the greatest cholesterol-lowering effects were seen in patients with initial total cholesterol values between 250 and 300 mg/dL. The results of this trial were somewhat contradicted by findings of a trial by Saradeth et al. [26] where 600 mg of dried garlic powder (Kwai, Lichwer standardized to 1.3% alliin) was given to healthy patients with normal lipid levels over a period of 10 weeks. There was a significant reduction in total cholestrol and triglycerides levels confirming the fact that it can induce changes in blood lipids, even if these variables had been normal to start with Similarly another trial by Gadkari and Joshi on healthy medical students after consumption of 10 gm raw garlic showed significant reduction in serum cholesterol and increase in clotting time and fibrinolytic activity [27].

Clinical trials using different types of garlic preparations in hypercholesterolemia patients have demonstrated debatable results, and it was assumed that these discrepancies may have resulted due to the differences of the composition of garlic preparations and the response they may induce. This fact was well proven by a study done by Sobenin et al. [28] in which patients with mildly raised lipid levels were given garlic powder tablets (allicor) containing 600 mg of garlic content. A moderate decrease in lipid levels was seen (7.6% decrease in cholesterol; 11.7% decrease in LDL levels); in addition, a substantial rise in HDL level 11.5% was also noticed. It was assumed that this hypocholesterolemic action of garlic preparations may be due to the use of a time-released form of garlic powder tablets. Similarly, a commonly used preparation of garlic in the form of AGE extract of 7.2 gm daily for 6 months also showed beneficial effects on the lipid profile of moderately hypercholesterolemia subjects. There was an overall 6.1% decrease in cholestrol levels and 4% decrease in LDL levels noticed thus confirming its efficacy [29]. 

Another randomized placebo control trial using 5 gm of raw garlic on patients with mildly raised lipids was used for 42 days and demonstrated significant reduction in cholestrol and triglyceride levels with a rise in HDL levels; however, these effects were not sustainable and returned to baseline levels as soon as the garlic use was withdrawn. This suggested that garlic consumption alone can decrease serum lipids in patients with mildly raised lipid levels; however, it cannot be used as the main therapeutic agent for hyperlipidemia [30]. 

Dyslipidemia refers to increase in cholestrol, triglycerides, and LDL levels with a decrease in HDL level (below 40 mg). It was expected that apart from decreasing cholestrol, LDL and triglycerides levels, garlic also has an impact on low HDL which was further established by a trial conducted on healthy individuals with a decreased HDL levels below 10 mg at baseline. They were given high-fat diet followed by garlic powder preparation (Sapec, Kwai) of 900 mg daily for 6 weeks. A significant decrease in triglyceride levels was observed in the treatment group in comparison to placebo group with a significant rise in HDL levels above baseline [31]. Similarly another study reported that 3 g of fresh garlic (1 clove) daily for 16 weeks had a 21% decrease in cholesterol levels [32].

Despite the existence of various clinical trials, the role of garlic in treating dyslipidemia is still debatable. In order to address this query a various meta-analyses were also conducted. A meta-analyses done by Silagy and Neil studied 16 trials among 952 patients using garlic, both in powder and nonpowder form. There was an overall reduction in cholestrol level seen that is, 8% with powdered form while 15% with nonpowder preparations. Significant lowering of serum triglyceride was also noticed, while HDL level remains unchanged [33].

Similarly, another meta-analyses by Warshafsky et al. among patients with cholestrol levels greater than 200 mg showed significant reduction in total cholesterol levels. It was suggested that garlic in an amount approximately one half to one clove per day is effective in reducing cholesterol levels by about 9% [34]. 

A recent meta-analyses conducted by Zeng et al. in 2012 clearly illustrated that garlic therapy is more effective if used for a long term with higher baseline total cholestrol levels; they also concluded that garlic powder and aged garlic extract were more effective in reducing serum TC levels, while garlic oil was more effective in lowering serum TG levels [35]. A trial comparing garlic with a commercial lipid-lowering drug (bezafibrate) found them to be equally effective in decreasing lipids to a statistically significant extent [36].

There were few clinical trials which did not show any effects on lipid levels. A trial done by Satitvipawee et al. for a period of 12 weeks using 5.6 mg/tablet garlic tablets showed no significant improvement in lipids levels [37].

 An RCT in which 900 mg garlic in the form of tablets (Kwai) was given daily to patients with hypercholesterolemia showed no significant change in lipid levels in comparison to placebo group [38]. Similarly, in other trial steams distilled garlic oil in a quantity of 5 mg twice daily for 12 weeks showed no influence on lipid levels [39]. A trial with garlic usage in the form of dried form in a dose of 600 mg to 1500 mg did not show any effects suggesting that dried preparation in the dosage studied were ineffective in reducing lipid levels [40]. Similarly, a meta-analyses by Khoo and Aziz also showed insignificant outcomes [41]. 

One trial of garlic extract treatment in children with hypercholesterolemia found no adverse effects and no significant beneficial effect on lipid levels [42].

Clinical investigations exploring the effects of garlic and its various preparations in hypercholesterolemia have demonstrated somewhat contradictory results. The diverse composition and amount of active sulfur compounds of different garlic preparations used in various trials might be responsible for the above mentioned inconsistent findings. Other factors like subject recruitment, duration of study, dietary control, lifestyle, and methods of lipid analyses may also have an influence. These findings emphasize the need for standardization of garlic preparations in order to reach to a valid conclusion.

## 12. Effects of Garlic on Hypertension

Hypertension is an important risk factor for leading to cardiovascular disease. Currently, it affects 1 billion people worldwide, and this number is expected to rise to 1.6 billion by 2025 [43, 44]. Garlic regular consumption has shown some association with blood pressure control. Blood pressure reducing properties of garlic are related with the hydrogen sulphide production [45] and allicin content liberated from alliin and the enzyme alliinase [46] which is assumed to possess angiotensin II inhibiting and vasodilating effects. Garlic is used as a treatment remedy by many people worldwide to control blood pressure. According to one survey, approximately 29% of people are using garlic for their blood pressure control [47].

The antihypertensive effects of garlic have been studied, but the remaining controversial various studies done showed controversial results as evident from Table 2. Clinical trial done by Zhang et al. consuming garlic oil in hypertensive patients over the 16-week period showed significant results [48]. A trial using garlic pearls containing 250 mg of garlic among hypertensive patients for 2 months demonstrated decrease in blood pressure level and also showed decrease in biomarkers responsible for oxidative stress in blood (plasma-oxidized LDL, plasma, and urinary concentration of 8-iso-Prostaglandin F2alpha) ultimately decreasing the risk of cardiovascular disease [49]. 

Majority of patients used garlic as a remedy for prevention from dyslipidemia and hypertension various illnesses. An RCT conducted by Ried et al. on patients with uncontrolled blood pressure used AGE preparation of 900 mg garlic containing (2.4 mg salicystine) for 12 weeks and concluded that significant reduction in blood pressure level was noted only among patients who had blood pressure values of more than 140 mm Hg at baseline [50] suggesting that its role in primary prevention is questionable.

Another trial by Duda et al. assessed the role of garlic on blood pressure and lipids levels and concluded that garlic can be used as a tentative treatment along with antihypertensive drug because of its positive effect on lipid levels and antioxidant properties [51].

Few meta-analyses were also done to see the efficacy. In 1994, a meta-analyses assessed the effect of garlic on hypertension, among which three trials showed significant reductions in systolic blood pressure (7.7 mm Hg greater reduction), and four trials showed reductions in diastolic blood pressure (5 mm Hg greater reduction) in comparison to placebo [52].

A meta-analyses conducted by Ried et al. showed significant results with decrease in systolic blood pressure of about 16.3 mm Hg and diastolic blood pressure of about 9.3 mm Hg in comparison to placebo group; however, these effects were only observed in patient having systolic blood pressure values more than 140 mm Hg [53]. 

Another meta-analyses done concluded that garlic reduces mean supine systolic and diastolic blood pressure by approximately 10–12 mm Hg and 6–9 mm Hg, respectively, over and above the effect of placebo, but the confidence intervals for these effect estimates are not clear cut, and this difference in blood pressure reduction may be due to subjective variation in blood pressure measurements suggesting more clinical trials [54].

Few trials done by Capraz et al. and Pittler and Ernst showed insignificant results [55, 56]. Similarly a meta-analyses done by Simons et al. also showed insignificant results with no effects on blood pressure levels and concluded that the effect of garlic on blood pressure cannot be established [57]. 

To ascertain the effectiveness of garlic in blood pressure reduction, very few studies are available which have shown small positive effects, insufficient to draw any conclusions. Information gathered from the previous meta-analyses is also inconclusive due to methodological shortcomings. Therefore, in our view, use of garlic cannot be recommended as antihypertensive advice for hypertensive patients in daily practice. Further, meta-analyses are required to prove its efficacy.

## 13. Effects on Platelets and Fibrinolytic Activity

Garlic has a beneficial effect on platelet adhesion or aggregation, a potential risk factor for cardiovascular disease. The self-condensation products of allicin and ajeones are said to have antithrombotic action, in addition to its potential effect in the inhibition of platelet aggregation [58] 23. Dissolution of clots and thrombi through fibrinolysis is also improved by garlic.

 A number of trials have been conducted to find out the usefulness of garlic or its preparation against platelets. A trial by Rahman and Billington reported that garlic causes inhibition of platelet aggregation by various mechanisms including inhibition of cyclooxygenase activity leading to thromboxane A2 formation, by suppressing mobilization of calcium into the platelets, and by increasing levels of messengers (cAMP and cGMP) with in the platelets. It also exhibits strong antioxidant property by increasing production of platelet-derived NO. Simultaneously, it also reduces the ability of platelets to bind to fibrinogen, thus overall resulting in inhibition of platelet aggregations and enhance fibrinolytic activity [59].

This fact was further confirmed by a trial by Allison et al. which showed that AGE extract modified raw preparation of garlic-inhibited platelet aggregation by suppressing the influx of calcium ions through their chelation within platelet cytosol or by altering other intracellular second messengers within the platelets [60]. 

A trial using AGE preparation of garlic recommended dose-dependent inhibition of platelet aggregation, that is, AGE inhibited platelet aggregation at dose of 7.2 gm however fibrinolytic activity was inhibited at all doses among hypercholesterolemia patients [61]. A trial on ischemic heart disease patients after using raw/fried garlic significantly increased fibrinolytic activity [62].

A study using garlic oil as an ingredient reported two important paraffinic polysulphides diallyl disulphide (DADS) and diallyl trisulphide (DATS) mainly responsible for causing antiplatelet inhibition. Action of DATS was found more potent as compared to DADS; however, it was seen that inhibition of platelet by DATS was reversible. The results of this trial conclude that garlic oil should not be used in patients with comorbid demanding necessary inhibition of platelets activity [63].

When discussing its efficacy in comparison to statins, its action was found comparable to compar to clopidogrel [64]. Similarly, it was also suggested that AGE preparation if taken as a dietary supplement by healthy individuals may be beneficial in protection against cardiovascular disease through inhibition of platelet aggregation [65].

All of the above results showed some beneficial effects; however, two studies done by [66] Legnani et al. and [67] Scharbert et al. on healthy individuals showed no effect on fibrinolysis and platelet activity.

It is concluded that garlic inhibits platelet aggregation by multiple mechanisms and may have a role in preventing cardiovascular disease. However, data is scarce, and further studies are required to prove this fact.

## 14. Garlic Role on Endothelium and Vascular Dilatation

Though garlic mainly protect against cardiovascular disease through reduction of lipid levels, however few studies suggest that it has some effects on endothelium and vascular dilatation through inhibition of oxidation process. Garlic contains allicin as the main active ingredient with prospect to provide beneficial effects on cardiovascular system. A study by Chan et al. [68] showed that allicin caused enhancement of antioxidant state by lowering of reactive oxygen species and increasing the production of glutathione. Similarly, garlic prevents from cardiovascular disease through inhibition of LDL oxidation thus inhibiting atherosclerosis of vessels, important risk factors for cardiovascular disease [69]. Budoff in 2006 conducted a pilot study in which patients who were already on statin therapy were given AGE extract of garlic and placebo and their degree of coronary artery calcification was assessed which slowed down in patient who were given Garlic therapy plus statin as compared to the other group [70].

Garlic role in primary and secondary prevention of cardiac disease was also questionable as few trials done showed positive results as demonstrated by Table 3. This fact was tested among patients with cardiovascular disease by giving garlic powder tablets allicor, and their 10-year prognostic risk of acute myocardial infarction and sudden death were assessed. It was seen that after 12-month treatment with allicor, there was significant decrease of cardiovascular risk. that is, 1.5 fold in men and 1.3 fold in women. The main influence that played a role in cardiovascular risk reduction was the decrease in LDL cholesterol by 32.9 mg/dL in men and by 27.3 mg/dL in women, thus proving the fact that it has effective role in secondary cardiovascular disease prevention [71].

## 15. Side Effects of Garlic

A couple of case reports have published the adverse effects of garlic ingestion, where one claimed allergic dermatitis observed in a patient taking raw garlic [72]. Another stated that the antithrombotic activity of garlic might interact with oral anticoagulants; therefore, caution must be taken when using in concordance with oral anticoagulants [73].

## 16. Conclusion

We conclude that the beneficial effect of garlic preparations on lipids and blood pressure extends also to platelet function, thus providing a wider potential protection of the cardiovascular system through its major effects on cholestrol reduction. However, its efficacy in blood pressure reduction is mild with some beneficial effects on platelet aggregation. This warrants the need for more meta-analyses using standardized preparations with a close watch on methodological shortfalls. 

## Figures and Tables

**Table 1:** Effects of garlic on lipid levels.

Study	Type	Target	Duration of Rx	Dose	Case/control	Outcome
Mader, (1990) [20]	Randomized, placebo-controlled trial	Hyperlipidemic	12 weeks	800 mg garlic powder	130/131	Dec in T. chol level—12%, TG level—17%

Gadkari and Joshi, (1991) [27]	Randomized control trial	Normal individuals	2 months	10 gm of raw garlic	25/25	Dec T. chol, increase clotting time, and fibrinolytic activity

Rotzsch et al., (1992) [31]	Randomized, placebo-controlled, double-blind trial	Healthy individuals with low HDL	6 weeks	900 mg garlic powder	12/12	Dec TG levels and increase HDL levels

Saradeth et al., (1994) [26]	Randomized double-blind study, placebo-controlled trial	Healthy individuals with normal lipid levels	15 weeks	600 mg dried garlic powder	34/34	T. chol dec from 223 to 214 mg/dL TG dec from 124 to 118 mg/dL

Steiner et al., (1996) [29]	Double-blind crossover trial	Hyperlipidemic	11 months	7.2 g aged garlic	20/21	Dec T. chol 6.1%, dec LDL 4%, systolic BP 5.5% dec, and modest dec in diastolic Bp noticed

Isaacsohn et al., (1998) [38]	Randomized, double-blind, placebo-control trial	Hyperlipidemic	12 weeks	900 mg garlic powder (Kwai)	28/22	No change in lipid levels noticed

Berthold et al., (1998) [39]	Double-blind, randomized, placebo-controlled trial	Hyperlipidemic	12 weeks	10 mg garlic oil	12/13	No change in lipids or lipoproteins levels noticed

Satitvipawee et al., (2003) [37]	Randomized, double-blind, placebo-controlled trial	Hyperlipidemic	4 weeks/12 weeks	Garlic extract	70/76	No dec in T. chol, DL, TG, and HDL levels noticed

Mahmoodi et al., (2006) [30]	Clinical trial	Hyperlipidemic	42 days	Raw garlic 5 gm twice daily	30	Dec T. chol, dec LDL, dec TG, increase HDL level Reversed after stopping of garlic

Sobenin et al., (2008) [28]	Double blinded placebo controlled	Hyperlipidemic	12 weeks	Allicor (600 mg daily)	21/21	T. chol 7.6% dec, LDL 11.8%, and HDL inc 11.5%

T. chol: total cholesterol, HDL: high-density lipoprotein, TG: triglyceride, LDL: low-density lipoprotein, and VLDL: very low-density lipoprotein.

**Table 2:** Effect of garlic on blood pressure levels.

Study	Type	Target	Duration of Rx	Dose	Case/control	Outcome
Zhang et al., (2001) [48]	Parallel-controlled trial	Hypertensives	16 weeks	Distilled garlic oil 12.3 mg/d	14/13	Garlic oil lowers SBP and DBP

Dhawan and Jain, (2004) [49]	Not placebo controlled	Hypertensives	2 months	Garlic pearls 250 mg	20/20	Dec Bp, dec ox-LDL, and 8-iso-PGF2alpha levels

Capraz et al., (2007) [55]	Randomized placebo control trial	Hypertensives	70 minutes	Rw garlic, Garlic tablets	25/25/25	No effects on BP levels

Duda et al., (2008) [51]	Prospective and uncontrolled clinical study	Hypertensives	30 days	Antihypertensive drug + Garlic capsules	38/32	Dec total lipids and lipid peroxidation noticed

Ried et al., (2010) [50]	Randomized placebo control trial	Hypertensives	12 weeks	960 mg AGE	25/25	SBP—10.2 ± 4.3 mmHg dec

Ried et al., (2013) [53]	Double-blind, randomized placebo-controlled, dose-response trial	Hypertensives	12 weeks	Aged garlic 240/480/960 mg	26/26/27	SBP—11.8 ± 5.4 mmHg, (2 capsule) 7.4 ± 4.1 mmHg (4 capsule)

T. chol: total cholesterol, HDL: high density lipoprotein, TG: triglyceride, LDL: low-density lipoprotein, VLDL: very low-density lipoprotein, 8-OHdG: (8-Hydroxy-2′-deoxyguanosine), and 8-iso-PGF2alpha: (8-iso-Prostaglandin F2alpha).

**Table 3:** Effect of garlic on cardiovascular disease.

Study	Type	Target	Duration of treatment	Dose	Route	Case/control	Outcome
Bordia et al., (1998) [63]	Placebo control trial	Coronary artery disease patients	3 months	1 gm garlic (capsules)	Oral	30/30	Dec T. chol and TG, increase HDL level, and no effect on fibrinogen and glucose level

Sobenin et al., (2010) [71]	Randomized control trial	Coronary artery disease patients	1 year	Time-released garlic powder	Oral	26/25	Dec LDL—32.9 mg/dL in males, 27.3 mg/dL in females

T. chol: total cholesterol, HDL: high-density lipoprotein, TG triglyceride, LDL: low-density lipoprotein.
